# Effects of Glucose Deprivation on ATP and Proteoglycan Production of Intervertebral Disc Cells under Hypoxia

**DOI:** 10.1038/s41598-020-65691-w

**Published:** 2020-06-01

**Authors:** Xue Yin, Aarif Motorwala, Oraya Vesvoranan, Howard B. Levene, Weiyong Gu, Chun-Yuh Huang

**Affiliations:** 1 Department of Biomedical Engineering, University of Miami, Coral Gables, FL USA; 2 Department of Neurological Surgery, University of Miami Miller School of Medicine, Miami, FL USA; 3 Department of Mechanical and Aerospace Engineering, University of Miami, Coral Gables, FL USA

**Keywords:** Nutrition, Cell biology, Mechanisms of disease

## Abstract

As the most common cause of low back pain, the cascade of intervertebral disc (IVD) degeneration is initiated by the disappearance of notochordal cells and progressive loss of proteoglycan (PG). Limited nutrient supply in the avascular disc environment restricts the production of ATP which is an essential energy source for cell survival and function such as PG biosynthesis. The objective of this study was to examine ATP level and PG production of porcine IVD cells under prolonged exposure to hypoxia with physiological glucose concentrations. The results showed notochordal NP and AF cells responded differently to changes of oxygen and glucose. Metabolic activities (including PG production) of IVD cells are restricted under the *in-vivo* nutrient conditions while NP notochordal cells are likely to be more vulnerable to reduced nutrition supply. Moreover, provision of energy, together or not with genetic regulation, may govern PG production in the IVD under restricted nutrient supply. Therefore, maintaining essential levels of nutrients may reduce the loss of notochordal cells and PG in the IVD. This study provides a new insight into the metabolism of IVD cells under nutrient deprivation and the information for developing treatment strategies for disc degeneration.

## Introduction

As a leading cause of global disability, low back pain (LBP) creates a significant social economic burden^1^. It is estimated to affect 2.06 million people at an incidence rate of 1.39 per 1,000 person-years in the United States^2^. About 80% of adult population is subjected to the disease and the likelihood peaks in the third decade of life^3,4^. LBP is closely related to degeneration of the intervertebral disc (IVD) which is formed by a specialized extracellular matrix (ECM) filled with water and sparsely distributed cells. IVD cells within the two anatomically different regions, namely nucleus pulposus (NP) and annulus fibrosus (AF), play an essential role in the anabolic and catabolic activities for ECM turnover^5^.

While the clinical consequences of IVD degeneration are well documented, the exact causes for IVD degeneration remain undetermined. Nutritional deprivation in the disc microenvironment is believed to be one of the potential causes for disc degeneration^6,7^. It is widely accepted that disappearance of notochordal NP cells and reduced content of proteoglycan (PG) (a key ECM component) correlate with degenerative changes of the IVD^8–10^. Since PGs provide the disc a necessary shock-absorbing ability to withstand mechanical forces through the interaction between their negatively charged sulphate groups and water^11^, the loss of PGs in degenerated disc was strongly associated with decreased water content^8^ which is a major component of the disc (70–90% of wet weight in NP and 60–70% in AF)^9,12,13^. As a result, degenerated disc tissues appear dehydrated and fibrotic with a blurred boundary between NP and AF regions^9,14^.

Cells rely on nutrient supply for survival and function. Since key nutrients such as glucose and oxygen can only be delivered to cells by diffusion through the dense ECM from capillaries at the endplate or the margins of AF due to the avascular nature of the IVD^15–17^, ECM transport properties and cellular consumption establish sharp concentration gradients of nutrients with lowest values at the disc center (i.e., NP region)^18–20^, which imposes challenges to maintain a healthy cell condition within the IVD. Nutrient concentrations have been demonstrated to exert profound impacts on cell viability and energy metabolism as well as matrix biosynthesis^20–22^. Glucose is one of the critical nutrients for survival of IVD cells^21^. A threshold value of 0.5 mM glucose has been previously suggested for NP cells to survive^21^.

Furthermore, one imperative role of IVD cells is their ECM biosynthesis to maintain disc integrity. The biosynthesis of PG utilizes a large amount of ATP to fuel its posttranslational modifications and form a sulfate donor, phosphoadenosine^23,24^. Therefore, ATP not only provides a direct energy source but also serves as a building block for PG biosynthesis. Anaerobic glycolysis, an inefficient way to produce ATP, has been suggested as the dominant pathway for ATP production of IVD cells within the hypoxic disc environment^15,17,21,25^. Depletion of intracellular ATP was implicated as a contributor for degeneration of articular cartilage^26,27^ which relies on glycolysis for ATP production as IVD cells. However, the energy metabolism of IVD cells under glucose deprivation and hypoxia and its association with disc degeneration have not been elucidated. The objective of this study was to examine the intracellular ATP content and PG production of IVD cells under prolonged exposure to hypoxia with a range of glucose levels representing the *in vivo* concentration gradient in the IVD.

## Results

### Viability of porcine IVD Cells

**Figure 1:**
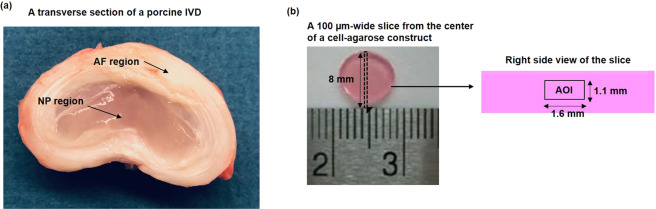
(**a**) A transverse section of a porcine IVD with harvesting sites indicated. (**b**) The location of the slice on agarose construct and the location of AOI on the slice for evaluation of cell viability.

**Figure 2:**
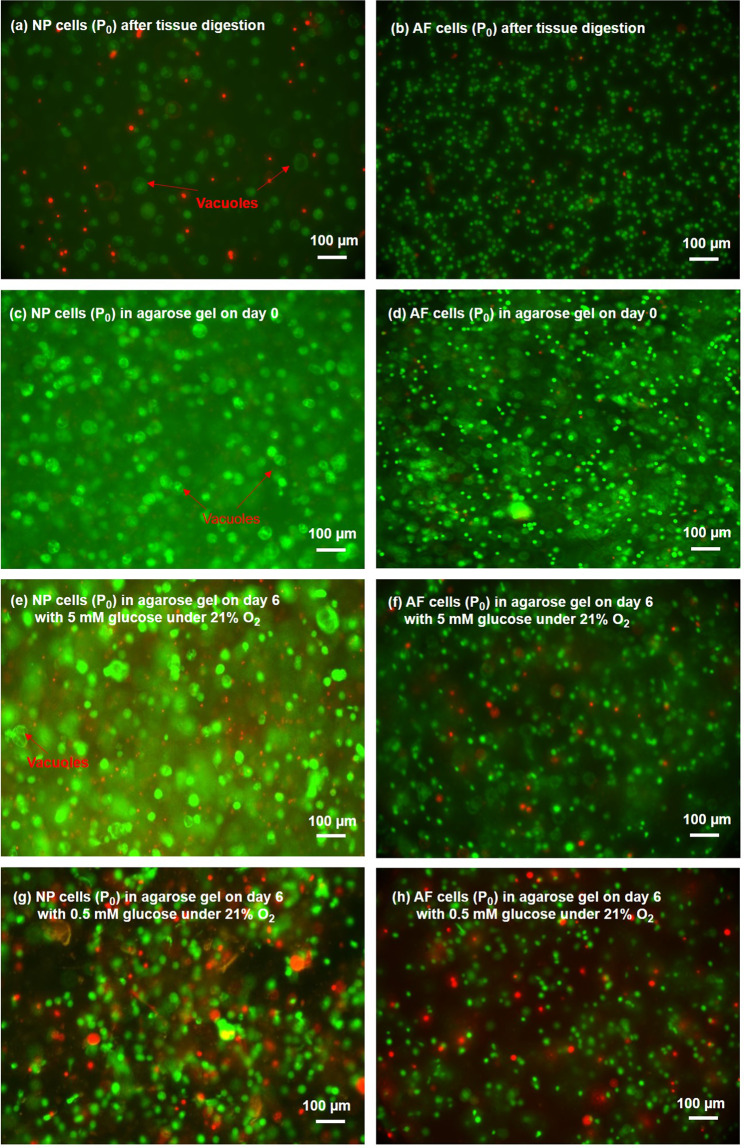
Typical Live/Dead staining of (**a,c,e,g**) NP cells and (**b,d,f,h**) AF cells after tissue digestion and in agarose on day 0 and day 6 (green/red: live/dead cells).

**Figure 3:**
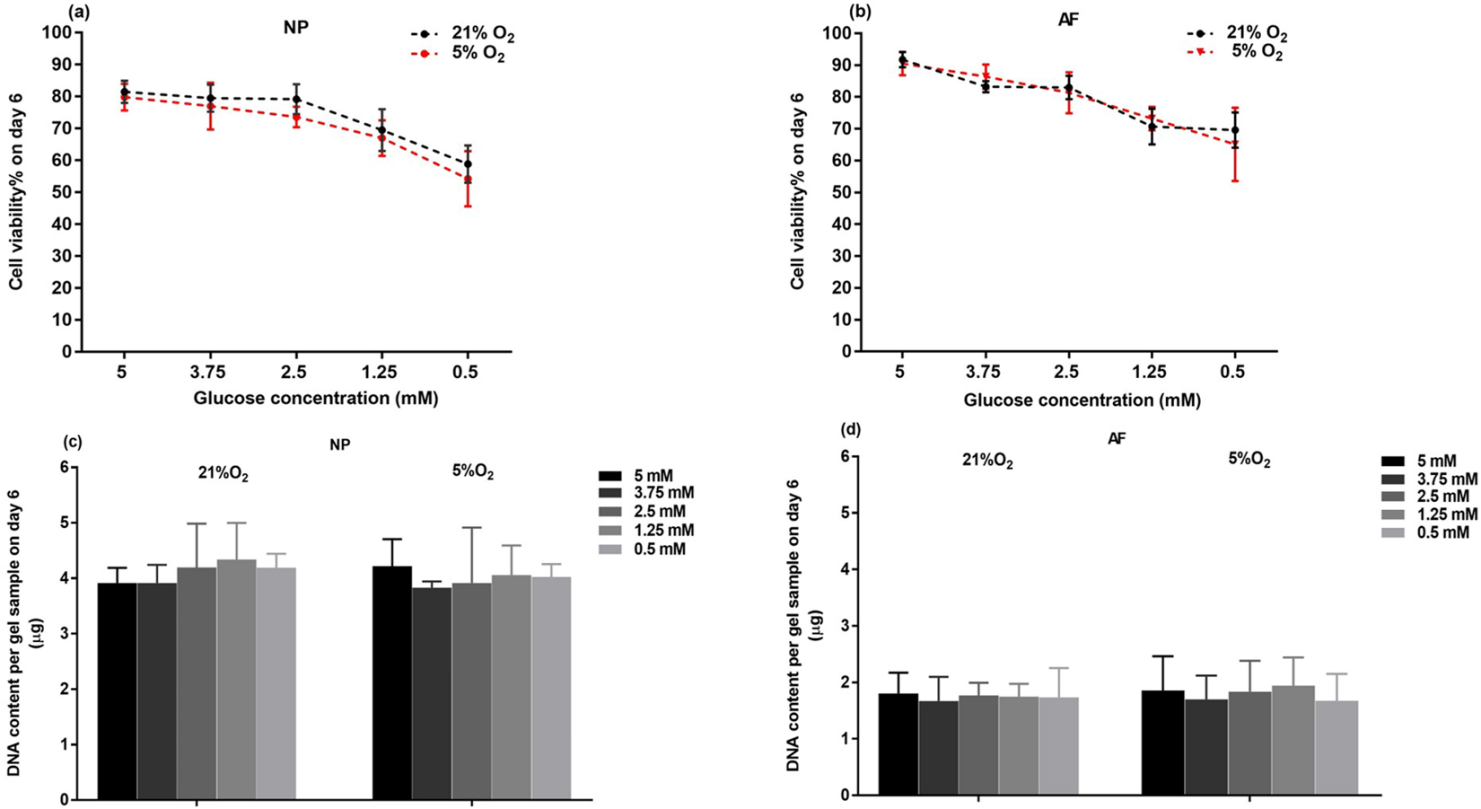
Viability and DNA content of (**a,c**) NP and (**b,d**) AF cells at various glucose concentrations under 21% and 5% O_2_ on day 6 (n = 9). For viability of NP cells under both 21% and 5% O_2_, 5 mM, 3.75 mM, 2.5 mM glucose > 1.25 mM > 0.5 mM (p < 0.05). For viability of AF cells under both 21% and 5% O_2_, 5 mM, 3.75 mM, 2.5 mM glucose > 1.25 mM, 0.5 mM glucose (p < 0.05). No statistical significances were found in DNA content among the different treatment groups for both NP and AF cells.

High cell viability of both NP and AF cells were observed after tissue digestion (Figs. 1, 2a,b) and after seeding in agarose constructs (day 0 of the experiment)(Fig. 2c,d). Both NP and AF cells remained alive at the construct center under all treatment conditions after 6 days of culture (Fig. 2e–h). Hypoxia did not significantly affect viability of both cell types at any glucose level (Fig. 3a,b). However, significant decreases in cell viability (~20%) on average was observed when the glucose concentration was reduced to 1.25 mM and 0.5 mM for both cell types (Fig. 3a,b). However, no significant differences were found in DNA content, an indicator for the total number of cells, among the glucose groups at the same oxygen level on day 6 for both cell types (Fig. 3c,d).

### Glucose consumption rate of porcine IVD cells

**Figure 4:**
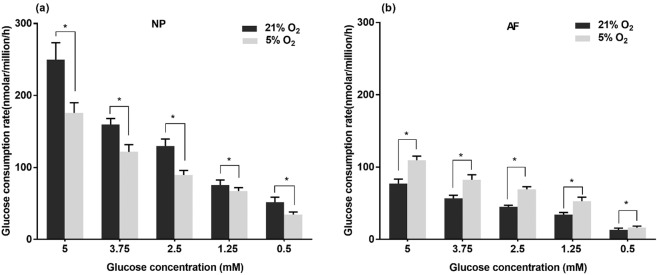
Comparison of glucose consumption rates of (**a**) NP and (**b**) AF cells over 3 days of culture at various glucose concentrations under 21% and 5% O_2_ (n = 9). For both NP and AF cells under 21% O_2_ and 5% O_2_: 5 mM > 3.75 mM > 2.5 mM >1.25 mM > 0.5 mM (p < 0.05). The * symbol indicates significant differences (p < 0.05) between 21% O_2_ and 5% O_2._.

The average glucose consumption rates from day 1 to day 3 and day 3 to day 6 were not significantly different among the experimental groups. Therefore, only the data measured over the first 3 days were presented. NP cells consumed more glucose than AF cells at the same nutrition level. The glucose consumption rates of both NP and AF cells significantly decreased with reducing glucose concentration for both oxygen levels (p < 0.05) (Fig. 4). Under hypoxia, NP cells consumed significantly less glucose (p < 0.05, ~1.36-fold decrease) whereas the glucose consumption rate of AF cells significantly increased (p < 0.05, ~1.44-fold increase) for each glucose level measured.

### Intracellular ATP content of porcine IVD Cells

**Figure 5:**
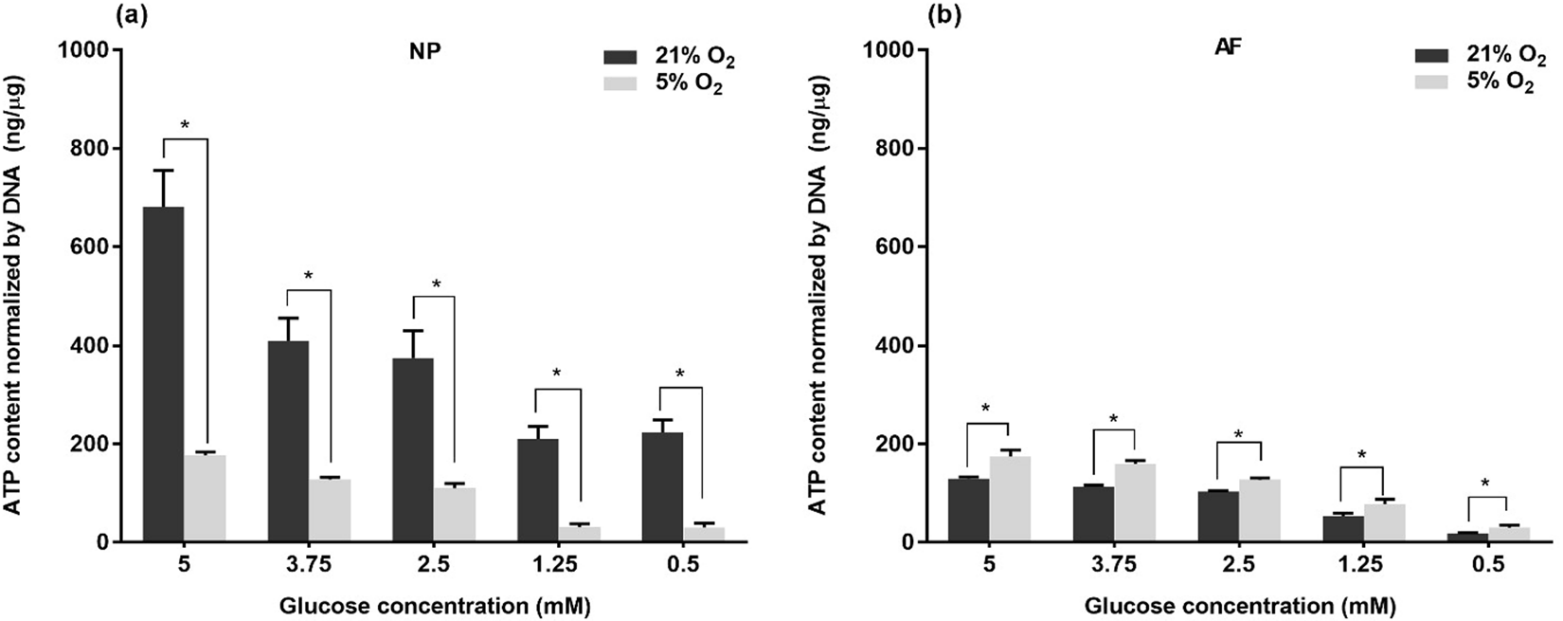
Comparison of intracellular ATP content of (**a**) NP cells and (**b**) AF cells at various glucose concentrations under 21% and 5% O_2_ on day 6 (n = 9). For NP cells under 21% O_2_: 5 mM > 3.75 mM, 2.5 mM >1.25 mM, 0.5 mM (p < 0.05). For NP cells under 5% O_2_: 5 mM > 3.75 mM > 2.5 mM >1.25 mM, 0.5 mM (p < 0.05). For AF cells both under 21% O_2_ and 5% O_2_: 5 mM > 3.75 mM >2.5 mM >1.25 mM > 0.5 mM (p < 0.05). The * symbol indicates significant differences (p < 0.05) between 21% O_2_ and 5% O_2._.

For each oxygen level, the ATP content of NP cells significantly decreased with reducing glucose supply (p < 0.05) when the glucose level was above 1.25 mM (Fig. 5a). However, no significant difference in ATP content of NP cells was found between the glucose levels of 0.5 mM and 1.25 mM at the same oxygen level (Fig. 5a). A glucose concentration-dependent decline in intracellular ATP content was also found in AF cells throughout all glucose concentrations for both oxygen levels (Fig. 5b). However, compared to normoxia, the ATP content of NP cells was significantly reduced (p < 0.05, ~ 4 folds) whereas a significant increase (p < 0.05, 1.42 folds) was found in the ATP content of AF cells for each glucose level under hypoxia.

### PG production of porcine IVD cells

**Figure 6:**
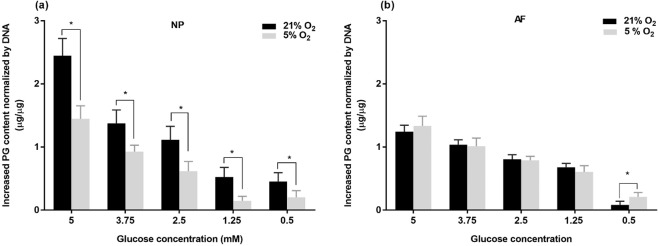
Comparison of the PG content of (**a**) NP cells and (**b**) AF cells at various glucose concentrations under 21% and 5% O_2_ on day 6 compared to day 0 (n = 9). For the NP cells under 21% O_2_: 5 mM > 3.75 mM, 2.5 mM >1.25 mM, 0.5 mM (p < 0.05). For NP cells under 5% O_2_: 5 mM > 3.75 mM > 2.5 mM >1.25 mM, 0.5 mM (p < 0.05). For AF cells both under 21% O_2_ and 5% O_2_: 5 mM> 3.75 mM >2.5 mM >1.25 mM > 0.5 mM (p < 0.05). The * symbol indicates significant differences (p < 0.05) between 21% O_2_ and 5% O_2_.

Similar to intracellular ATP content, a reduction of glucose supply significantly decreased the PG production of both NP and AF cell types at each oxygen level (Fig. 6) except that there were no significant differences in PG production of NP cells between the glucose levels of 0.5 mM and 1.25 mM (Fig. 6a). Hypoxia significantly reduced the PG production of NP cells (p < 0.05; 2.14 folds) (Fig. 6a) while did not change or slightly upregulated the PG production of AF cells for each glucose level (Fig. 6b).

### Comparison between NP and AF cells

Under normoxia, NP cells exhibited significantly higher glucose consumption, the ATP level and PG production than AF cells for all glucose levels (p < 0.05 for all glucose levels) (Figs. 4–6). Under hypoxia, the glucose consumption rate of NP cells was significantly higher than that of AF cell for all glucose levels (Fig. 4), whereas AF exhibited a higher ATP content at the glucose levels of 3.75 mM (p < 0.05), 2.5 mM (p < 0.05) and 1.25 mM (p < 0.05) (Fig. 5) and a higher PG production at the glucose levels of 2.5 mM (p < 0.05) and 1.25 mM (p < 0.05) compared to NP cells (Fig. 6).

### Correlation of intracellular ATP and PG production of porcine IVD cells

**Figure 7:**
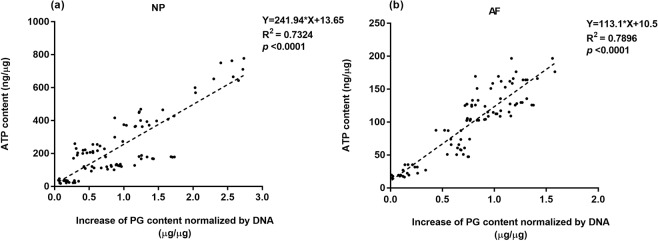
Correlation of PG biosynthesis and intracellular ATP content of (**a**) NP and (**b**) AF cells on day 6.

Regression analysis demonstrated a significant correlation (p < 0.001) between the PG production and ATP content for both NP and AF cells under all treatment conditions with the intracellular ATP content positively correlating with the PG production (Fig. 7).

### Gene expression of porcine IVD cells

**Figure 8:**
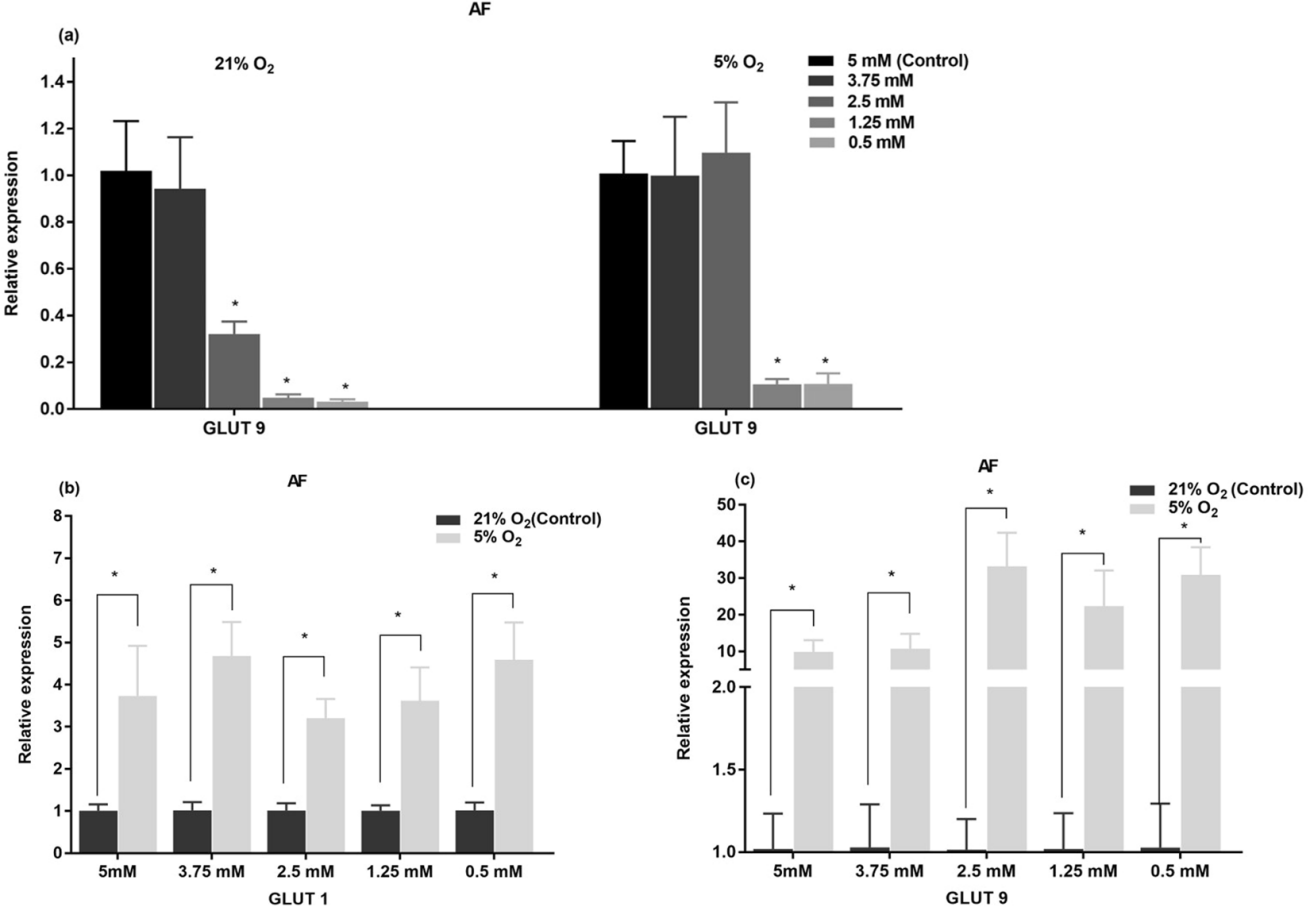
(**a**) Effects of glucose concentration on gene expression of GLUT-1 and GLUT-9 of AF cells under 21% O_2_ and 5% O_2_. Comparison of gene expression of (**b**) GLUT-1 and (**c**) GlUT-9 of AF cells between 21% O_2_ and 5% O_2_ for individual glucose levels. All data were normalized by their respective control group. The * symbol indicates significant differences compared to the control group (p < 0.05).

Glucose and oxygen levels did not significantly affect the gene expression of aggrecan and GLUT-3 for both cell types and gene expression of GLUT-1 and -9 of NP cells. However, for AF cells, the gene expression of GLUT-9 was significantly decreased with reducing glucose level (Fig. 8a) whereas the gene expression of GLUT-1 was independent of glucose level at the same oxygen level. Hypoxia significantly upregulated the gene expression of GLUT-1 and GLUT-9 in AF cells by 3–5 folds and 10–40 folds, respectively (Fig. 8b,c).

### Glucose concentration in degenerated human NP tissue

The density and glucose concentration of human NP tissues were measured as 1.04 ± 0.006 g/mL and 0.603 ± 0.108 mM, respectively.

## Discussion

The goal of this study was to investigate the ATP metabolism of IVD cells in response to a reduced supply of oxygen and glucose and its relationship with cell survival and PG production. To achieve this, we investigated intracellular ATP content, PG production, cell viability, glucose consumption rate and gene expression of facilitative glucose transporters and aggrecan of IVD cells cultured in a sequential reduction of glucose under both normoxia and hypoxia for 6 days. Due to limited availability of human tissues, porcine IVD tissues were used in this study in order to provide a sufficient amount of IVD cells, especially notochordal cells^28,29^ that vanish in adult IVDs. The results of this study demonstrated that NP (notochordal) and AF (chondrocyte-like) cells adapted differently to nutritional changes while oxygen and glucose supplies significantly affected ATP content and PG production of both cell types.

Under normoxia, NP cells exhibited significantly higher glucose consumption, ATP content and PG production compared to AF cells, suggesting notochordal NP cells are more metabolically active^21,30^. However, hypoxia dramatically impaired glucose uptake, intracellular ATP level and PG biosynthesis of NP cells whereas AF cells behaved in a different manner with increased glucose uptake and intracellular ATP. These findings indicate that notochordal NP cells may be more vulnerable to nutritional stress, especially under hypoxia. Although aerobic glycolysis is the main mechanism of energy production in IVD cells^15,17,21,25^, both NP and AF cells consume a significant amount of oxygen^21,31^ that creates a steep oxygen gradient within the IVD^32,33^. In addition to postnatal vascular regression^34^, disc size increases more than 3 folds from birth to adolescence^35,36^, considerably restricting the transport of nutrients into the center of the IVD^37^. In a normal adult disc, the oxygen level could reduce from 10% at the vascularized regions (i.e., outer annulus and the disc-bone junction) to 1% at the disc center^32,33^. Thus, substantial nutrient reduction may cause the disappearance of notochordal cells during adolescence^38,39^. Since AF cells are more tolerant to reduction of glucose and oxygen supplies, AF cells (chondrocyte-like cells) may be more capable of adapting to the harsh environment in the disc center than notochordal NP cells.

Previous theoretical studies demonstrated that the glucose level can be as low as 0.5 mM in degenerative adult discs^33,40^, which is consistent with the finding that the glucose concentration in the NP tissues obtained from human degenerative discs was 0.6 mM on average. The threshold of glucose level for survival of IVD cells has been suggested as 0.5 mM^21^, which is supported by our finding that the viability of both NP and AF cells remained high at all glucose levels after 6 days of culture. It suggests that the glucose level (i.e., 0.5 mM) in degenerative discs may be sufficient for maintaining viability of NP cells at the disc center. Indeed, no age-dependent changes in cell density of human IVDs were observed after adolescence^39^ whereas disc degeneration positively correlated with age^41^. Since previous studies have demonstrated that NP cells isolated from human degenerative discs were able to proliferate and produce PG with adequate nutrient supply under *in-vitro* culture^42–44^, increasing nutrient supplies may help mitigate disc degeneration by promoting metabolic activities of NP cells, such as ECM biosynthesis.

The present study found that under hypoxia, the ATP content and PG production of NP cells abruptly decreased 72% and 76%, respectively, when the glucose level dropped from 2.5 mM to 1.25 mM, and then remained constant at the glucose levels of 1.25 mM and 0.5 mM in despite of a significant decrease in glucose consumption rate (~43%) from 1.25 mM to 0.5 mM. Since PG biosynthesis is an energy demanding process, when the glucose level reaches the minimum level required for survival (i.e., 0.5 mM [20]), IVD cells may conserve energy for maintaining vital functions by downregulating or even shutting down high energy-consuming activities, a strategy commonly shown in aggressively growing cancer cells under nutrient starvation^45,46^. Our finding showed that when the glucose level was reduced below 2.5 mM, a significant decrease in cell viability was observed. It suggests that the ATP content reaches the minimum essential level for survival of NP notochordal cells whose metabolic activities (including PG production) have been restricted. Similarly, a substantial reduction in the ATP content (~62%) and PG production (~66%) of AF cells was also observed from 1.25 mM to 0.5 mM. Previous theoretical studies showed that the glucose level could be less than 1.25 mM^32,33^ in the NP of normal human IVDs and further reduced due to several factors such as calcified endplates and diminished vertebral blood supply^6,32,33^, indicating that the glucose level in the NP of the adult IVDs may be below a threshold (e.g., 1.25 mM) required for maintaining a normal PG content in the IVD. It could be the reason that PG content in human IVDs decreases with increasing age from young adulthood^8^. Taken together, the findings of this study support the observation that degenerative changes of the IVD begins as early as adolescence and worsens with increasing age^41^.

The finding of this study also indicates that NP cells may have initiated protective mechanisms to maintain a vital ATP level when the glucose level dropped below 2.5 mM. One possible mechanism is to recycle scavenged intracellular components via autophagy^47–49^. Indeed, autophagy has been recently demonstrated in human IVD cells as an early response to glucose withdrawal^50^ which could be initiated through activation of AMP-activated protein kinase (AMPK) pathway, a highly conserved energy sensor commonly triggered by low intracellular ATP level^51–54^. Another mechanism for cells to resist metabolic stress is to utilize amino acids as an alternative energy source^55–57^. For instance, as the most abundant free amino acids circulating in plasma^58^, glutamine is a ready source of carbon and nitrogen to support biosynthesis of nucleotides and amino acids^59,60^. It is well documented that rapidly proliferating cancer cells satisfy their high energy demands by compensating their aerobic glycolysis with glutaminolysis under hypoxia^61,62^.

NP cells contain functional mitochondria^21,63^, which makes aerobic respiration possible for a more efficient ATP production in the presence of oxygen. In fact, utilization of oxidative phosphorylation for ATP generation by NP notochordal cells have been evidenced in the study of Guehring *et al*.^21^. The current finding that hypoxia significantly reduced intracellular ATP content of NP cells at all glucose concentrations also lends support to this hypothesis. In contrast, the finding that hypoxia increased the ATP level and glucose consumption of AF cells is consistent with the study of Ishihara and Urban^20^, suggesting that AF cells exhibit a positive Pasteur effect which may be induced by the activation of the hypoxia inducible factor-1 (HIF-1)^64^.

Glucose is transported across the cell membrane by ubiquitous glucose facilitative transport systems^65^. Up to 14 isoforms of GLUT proteins have currently been identified in humans^66^. Expression of GLUT-1, −3 and −9 was detected in human IVD cells and affected by disc degeneration^67^. We found that hypoxia produced distinct effects on expression of GLUT-1, −3 and −9 in NP and AF cells. Upregulation of glucose transporters (GLUT-1 and −9) expression in AF cells by hypoxia (Fig. 7) is consistent with increases in ATP level and glucose consumption, suggesting glycolysis is stimulated by hypoxia in AF cells. HIF-1α signaling pathway was suggested to play a major role in mediating the gene expression of GLUT-1 under hypoxia^68,69^. Furthermore, this is the first study demonstrating that the GLUT-9 gene expression of AF cells is highly sensitive to both glucose and oxygen levels, indicating that GLUT-9 may play a significant role in energy metabolism of AF cells under nutrition deficiency. However, this study found no significant effects of hypoxia on the expression of three GLUT isoforms in NP cells, which is in agreement with the results from the study of Agrawal *et al*.^63^. It could be due to stable expression of HIF-1α in NP cells under both normoxic and hypoxic conditions^63^. Nevertheless, the current study also found that hypoxia significantly reduced the glucose consumption and ATP content of NP cells at all glucose concentrations (Fig. 4a), indicating that glycolysis was not stimulated by hypoxia as seen in AF cells. Further investigation is needed to elucidate the reasons why hypoxia reduced the glucose consumption of NP cells.

Our results provide the first evidence of a strong correlation between the PG production and intracellular ATP level of IVD cells (Fig. 6). However, the current study showed that gene expression of aggrecan remained constant for all nutrient conditions, suggesting the provision of energy rather than genetic regulation governs PG production in the IVD under restricted nutrient supply.

Several methods have been proposed to promote nutrient transport and energy production in the IVD^32,70–72^, especially dynamic compression that can enhance not only anabolic gene expression^73^ but also energy production in the IVD^72,74^. Furthermore, besides PG production, protein synthesis is also one of the most energy costly processes^45^. Nutrient deprivation may affect synthesis of cell signaling proteins, altering cell function^75^. Therefore, enhancing transport of nutrients into the IVD by dynamic compression or other approaches may help NP notochordal cells to remain normal functioning and produce a sufficient amount of PGs.

There are several limitations in present study. The first is that the hypoxic effect on the ATP and PG production of IVD cells was only examined under 5% oxygen. However, the physiological oxygen condition in the center NP can be as low as 1% in avascular IVD^18^. Ishihara and Urban found different effects of oxygen on energy metabolism of IVD cells as O_2_ was reduced from 21% to 5% compared to from 5% to 1%^20^. Therefore, since the oxygen level in the IVD could be as low as 1%, it will be interesting to carry out further investigations on the ATP content and PG production of IVD cells under the oxygen concentrations lower than 5%. Secondly, the mechanical environment of the IVD was not considered within the scope of this study. Since our lab previously found dynamic compression promoted ATP production and release of NP and AF cells^30,72^, the combined effects of nutrition and mechanical loading can represent *in vivo* conditions more closely. The third limitation is that DMEM used in the present study contained high concentrations of essential amino acids and vitamins, which may not represent the *in vivo* nutrient condition in the IVD. Since amino acids may be an alternative energy source for cells, we expected that the ATP level and PG production of IVD cells may be further reduced with fewer supplements of amino acids as the *in vivo* condition. Furthermore, this study did not take into account the acidic condition in the IVD that may reduce the metabolic rate of IVD cells^20^. In addition, the experimental culture conditions used in this study were different from the *in-vivo* environment of IVD cells, which may affect the metabolism of IVD cells. For instance, increased cell-cell interactions in cell clusters in the notochordal nucleus pulposus may elicit paracrine signalling and alter survial and functionality of NP cells^76^. *In-vivo* ECM rich enviroment may promote the interation between ECM and IVD cells^77^. Therefore, further studies may be carried out to elucidate the effects of cell density and ECM on metabolic responses of IVD cells. Lastly, the collagen content of the IVD is known to be varied with disc degeneration^8^. It will be worth of exploring the association between energy metabolism and collagen production under nutrient limitation.

In conclusion, this study demonstrated that intracellular ATP level of IVD cells strongly correlated with their PG production under the physiological levels of oxygen and glucose. A fall of either glucose or oxygen significantly reduced glucose uptake and intracellular ATP production of NP notochordal cells whereas AF cells exhibited an opposite response to hypoxia. In comparison to cell viability, our study suggests a higher threshold of glucose level (i.e., 2.5 mM) may be required for normal PG biosynthesis of NP notochordal cells. Therefore, maintaining essential levels of nutrients may reduce loss of notochordal cells and PG in the IVD and consequently slow down the development of disc degeneration. This study provides a new insight into the metabolism of IVD cells under nutrient deprivation and the information for developing treatment strategies for disc degeneration.

## Materials and Methods

### IVD cell culture and treatment

IVD tissues were harvested from spines of Yorkshire pig cadavers within 2 h of death (Male, 14–16 weeks, 80–100 lbs) acquired from the University of Miami Department of Veterinary Resource (Miami, FL) Tissue Sharing program (Institutional Animal Care and Use Committee approved source). The tissues obtained from NP or AF region (Fig. 1a) were pooled in high glucose (25 mM) Dulbecco’s Modified Eagle Medium (DMEM; Invitrogen Corp., Carlsbad, CA) containing 1 mg/ml type II collagenase (Worthington Biochemical Corp., Lakewood, NJ) and 0.6 mg/ml protease (Sigma Chemical, St. Louis, MO) for 24 h at 37 °C, 5% CO_2_. After enzymatic digestion, the cell-enzyme solution was filtered through a 70 μm strainer (BD Biosciences, San Jose, CA). IVD cells were isolated by centrifugation and re-suspended in DMEM supplemented with 10% fetal bovine serum (FBS; Invitrogen Corp., Carlsbad, CA) and 1% antibiotic-antimycotic (Invitrogen Corp, Carlsbad, CA). All freshly isolated notochordal NP cells were seeded into three-dimensional (3D) agarose gel (Low gelling temperature, Sigma Chemical, St. Louis, MO) with an established protocol^78–81^ to prevent phenotypic changes as suggested in literature^22,82,83^. Based on our previous study^84^, AF cells isolated from the same region expressed aggrecan and type II collgen, indicating a chondrocyte-like phenotype. The agarose culture was also used for AF cells as they tend to loss the chondrocyte-like phenotype in momolayer culture over time^84^. Each agarose construct contained 10^6^ cells in 2% agarose disk with a size of 8 mm in diameter and 2 mm in thickness (Fig. 1b). Although the cell density used in this study (10 million cells /mL) is slightly higher than the reported values of human IVDs *in vivo* (up to 9 million cells /mL)^5^, it allowed to obtain a sufficient amount of RNA from half of each sample for gene expression analysis. Following an initial incubation in DMEM containing 10% FBS and 1% antibiotic-antimycotic for 48 h, the agarose constructs were divided into 10 groups and cultured in 6 mL serum-free DMEM with various glucose concentrations (5 mM, 3.75 mM, 2.5 mM, 1.25 mM, 0.5 mM) in 5% O_2_ (hypoxia) or 21% O_2_ (normoxia) over 6 days. The media containing various glucose concentrations were made by a serial dilution of 25 mM DMEM with glucose-free DMEM and was stored at 4 °C until usage. Serum was excluded in present study to avoid other glucose sources and to provide a defined nutrition environment for examining the roles of oxygen and glucose in energy production. The hypoxic condition in this study was produced in a glovebox (LC-150 Stand Alone Glovebox system, LC Technology Solutions Inc., Salisbury, MA) modified by adding an oxygen controller (BioSpherix P110, Parish, NY) for maintaining 5% O_2_ level and a custom-made device for controlling the CO_2_ level at 5% using a K30 CO_2_ sensor (CO2Meter, Ormond Beach, FL). Cell experiments were performed in an incubator (GALAXY 48 R, VWR, Radnor, PA) that was placed inside the glovebox for maintaining proper humidity and temperature. Culture media were changed on day 3 for all groups. Media were equilibrated to the same O_2_ concentration for 24 h before being transferred to cell culture.

### Assessment of cell viability

To confirm cell survival under each treatment condition, cell viability was assessed on day 3 and 6. Briefly, a 100 µm-wide slice was cut from the center of the agarose gel construct (Fig. 1b) and incubated in phosphate buffered saline (PBS) containing 0.5 µL/mL calcein AM (4 mM) and 2 µL/mL ethidium homodimer-1 (2 mM) under room temperature for 30 min to allow the dyes to penetrate the cells according to the protocol in the Viability/Cytotoxicity Kit (Biotium, Fremont, CA). After the excessive dyes were removed by washing in PBS, an area of interest (AOI) with a dimension of 1.6 mm by 1.1 mm at the center of each slice was imaged under an inverted fluorescent microscope (Olympus IX70, Center Valley, PA). Approximately 200 cells within the AOI were counted in total for both cells types. Cell viability was quantified using Image J software.

### Measurement of average glucose consumption

Glucose uptake by IVD cells from day 1 to day 3 and from day 3 to day 6 was determined by measuring glucose depletion of culture medium. On day 3 and 6, 10 µL of culture medium was mixed with 90 µL of assay reagent from the Glucose (HK) Assay Kit (Sigma-Aldrich, St. Louis, Mo) followed by incubation for 15 min at room temperature according to manufacturer’s instructions. The absorbance was measured at 320 nm by a multimode plate reader (Beckman Coulter DTX 880, Brea, CA). Fresh culture media with different glucose concentrations (5 mM, 3.75 mM, 2.5 mM, 1.25 mM, 0.5 mM) served as standards. The average glucose consumption rates were calculated by normalizing glucose reduction by the number of live cells and time.

### Gene expression of aggrecan and GLUT-1, -3 and -9

The gene expression of aggrecan and glucose transporters (GLUT-1, -3, and -9) in IVD cells was analyzed by real-time RT-PCR. Two milliliters of Tri-Reagent (Molecular Research Center, Cincinnati, OH) were used to extract total RNA of each agarose construct according to manufacturer’s instructions. RNA pellets were dissolved in 20 μl of DNase/RNase free water and frozen at −80 °C overnight. On the next day, the RNA pellets were thawed and homogenized with a pellet pestle (Fisher Scientific, Waltham, MA) and centrifuged at 9000 rpm for 20 min at 4 °C in order to separate agarose from RNA. The supernatant containing RNA was collected and quantified by Qubit RNA HS assay kit (Life Technologies, Carlsbad, CA). Two hundred nanograms of total RNA were reverse-transcribed to cDNA using the High Capacity cDNA Reverse Transcription Kit (Applied Biosystems, Grand Island, NY). Real-time PCR (StepOnePlus, Applied Biosystems, and Grand Island, NY) was performed to analyze gene expression of aggrecan and GLUT-1, 3, 9. The results were normalized by 18 s gene as the endogenous control and calculated as relative expression to respective control group using the ${2}^{{-\Delta \Delta {\rm{C}}}_{{\rm{T}}}}$ method. For the study of the effect of glucose levels, the 5 mM glucose group served as the control for each oxygen level. Similarly, the measurements at 21% O_2_ were used as the control for examining the effect of oxygen level. The primer sequences used in this study are summarized in Table 1.

**Table 1:** Quantitative Real-Time PCR Primers.

Primer	Sequence	Size
**Aggrecan**	*Forward: AGACAGTGACCTGGCCTGAC*	151 bp
	*Reverse: CCAGGGGCAAATGTAAAGG*	
**18 s**	*Forward: CGGCTACCACATCCAAGGA*	188 bp
	*Reverse: AGCTGGAATTACCGCGGCT*	
**GLUT-1**	*Forward: GCTTCCAGTATGTGGAGCAACT*	132 bp
	*Reverse: AAGCAATCTCATCGAAGGTCC*	
**GLUT-3**	*Forward: GCGGCTGCCTTATGGGATTCT*	501 bp
	*Reverse: TGATGGGTTGCCGGTAGTTGG*	
**GLUT-9**	Forward: *GCCAGAGGAGAAAACTGGCT*	207 bp
	Reverse: GCTGCCACGGTAGATTAGCA	

### Biochemical analysis of PG production

On day 6, one half of each agarose construct was used to examine for PG content. Each sample was placed in 500 µL papain buffer (pH 7.4) containing 250 µg/ml papain (Sigma-Aldrich, St. Louis, Mo), 100 mM sodium phosphate, 10 mM EDTA and 10 mM cysteine HCl and incubated at 60 °C for 16 h. The amount of sulfated glycosaminoglycan in the digestion solution was determined by Dimethylmethylene (DMMB) blue dye-binding assay. Briefly, 20 µL aliquot of the papain digest was mixed with 200 µL of DMMB staining solution (pH = 1.5)^85^. Absorbance of the mixture was read at 525 nm by the multimode plate reader. Chondroitin sulfate from bovine trachea (Sigma-Aldrich, St. Louis, Mo) dissolved in the same papain buffer was used as the standard. All measurements were normalized by DNA content as determined by the Hoechst 33258 fluorometric assay^86^. The PG production was defined as an increased amount of PG content over 6 days by comparing with the PG content of agarose constructs on day 0.

### Measurement of intracellular ATP content

On day 6, ATP from the other half of each agarose construct was extracted by the Phenol–TE method^87^. Each agarose gel sample was homogenized (PowerMax AHS200, VWR, Radnor, PA) in 500 µL of water saturated phenol (Sigma-Aldrich, St. Louis, Mo) for 2 min. Five hundred microliters of chloroform and 250 µL of sterile water (VWR, Radnor, PA) were then added to the mixture. After centrifugation at 14,000 rpm for 10 min at 4 °C, the supernatant was removed and diluted 100 folds for NP samples and 20 folds for AF samples with nuclease-free water before being assayed for ATP content using the ATP Bioluminescent Assay Kit (Sigma-Aldrich, St. Louis, Mo) according to manufacturer’s instructions. ATP standard solutions were processed via the same extraction procedure before being used for measurement. The signal from pure water was used as control. The ATP content of each sample was normalized by its DNA content.

### Measurement of glucose concentration in degenerated human NP tissues

The study of human subjects was approved and by University of Miami Institutional Review Board and conducted according to its guidelines. Human NP tissues were surgically removed from degenerated lumbar IVDs of three patients (a 71 year female, a 55 year male and a 64 year female) and immediately transported to the laboratory. All patients signed the informed consent form for using their disc specimens in research. A sample of 50 ~ 60 mg was obtained from each tissue and digested in 500 µL PBS solution containing 250 µg/ml of papain (Sigma-Aldrich, St. Louis, Mo) and 10 mM of EDTA at 60 °C for 16 h^88^. The glucose concentration in the tissue digestion solution was measured using the Glucose (GO) Assay Kit (Sigma-Aldrich, St. Louis, Mo) according to manufacturer’s instruction. Briefly, 50 µL of the tissue digestion solution were mixed with 100 µL of the assay reagent and incubated at 37 °C for 30 min. Following addition of 100 µL sulfuric acid (VWR, Radnor, PA), the absorbance of the final mixture was measured at 540 nm by the multimode plate reader. The glucose concentration in each sample was calculated by dividing the glucose content of the sample by the volume of the sample that was calculated using the density of human IVD tissue determined using the buoyancy method as described in^89^. Briefly, tissue specimens were weighed in air (*Wt*) and in PBS (*W*_*PBS*_) using a density determination kit with an analytical balance (Sartorius, Germany). The volume of tissue sample was determined by dividing the buoyancy force by the mass density of PBS (${\rho }_{{PBS}}$ = 1.005 g/ml) according to the Archimedes’ principle. The tissue density ${\rho }_{t}$ (g/ml) was calculated as shown below:

$${\rho }_{t}=\frac{{W}_{t}}{({W}_{t}-{W}_{PBS})}{\rho }_{PBS}$$

Each experiment was performed in triplicate with each donor.

### Statistical analyses

All measurements were obtained from 3 independent experiments with one pig being used for each experiment. The results from a total of 9 samples for each experimental group (3 samples per group from each experiment) were pooled for statistical analyses. All results were presented as mean ± standard deviation. One-way ANOVA with Turkey’s HSD *post hoc* test (SPSS Statistics 20, Chicago, IL) was performed to compare cell viability, DNA content, glucose consumption rate, intracellular ATP content, gene expression of aggrecan and glucose transporters and PG production among groups with different glucose concentrations under the same oxygen level for each cell type. Unpaired two-tailed Student’s t-test was carried out to analyze differences in the measurements between two different oxygen levels at each glucose level for individual cell type or between two cell types for the same nutrient condition. Statistical significance was considered with p-value <0.05 in all analyses.
